# Improved Efficacy of Oral Immunotherapy Using Non-Digestible Oligosaccharides in a Murine Cow’s Milk Allergy Model: A Potential Role for Foxp3+ Regulatory T Cells

**DOI:** 10.3389/fimmu.2017.01230

**Published:** 2017-09-29

**Authors:** Marlotte M. Vonk, Mara A. P. Diks, Laura Wagenaar, Joost J. Smit, Raymond H. H. Pieters, Johan Garssen, Betty C. A. M. van Esch, Léon M. J. Knippels

**Affiliations:** ^1^Faculty of Science, Department of Pharmacology, Utrecht Institute for Pharmaceutical Sciences, Utrecht University, Utrecht, Netherlands; ^2^Immunology Platform, Nutricia Research, Utrecht, Netherlands; ^3^Faculty of Veterinary Medicine, Department of Immunotoxicology, Institute for Risk Assessment Sciences, Utrecht University, Utrecht, Netherlands

**Keywords:** cow’s milk allergy, oral immunotherapy, desensitization, non-digestible oligosaccharides, regulatory T cell, butyric acid, galectin-9

## Abstract

**Background:**

Oral immunotherapy (OIT) is a promising therapeutic approach to treat food allergic patients. However, there are some concerns regarding its safety and long-term efficacy. The use of non-digestible oligosaccharides might improve OIT efficacy since they are known to directly modulate intestinal epithelial and immune cells in addition to acting as prebiotics.

**Aim:**

To investigate whether a diet supplemented with plant-derived fructo-oligosaccharides (FOS) supports the efficacy of OIT in a murine cow’s milk allergy model and to elucidate the potential mechanisms involved.

**Methods:**

After oral sensitization to the cow’s milk protein whey, female C3H/HeOuJ mice were fed either a control diet or a diet supplemented with FOS (1% w/w) and received OIT (10 mg whey) 5 days a week for 3 weeks by gavage. Intradermal (i.d.) and intragastric (i.g.) challenges were performed to measure acute allergic symptoms and mast cell degranulation. Blood and organs were collected to measure antibody levels and T cell and dendritic cell populations. Spleen-derived T cell fractions (whole spleen- and CD25-depleted) were transferred to naïve recipient mice to confirm the involvement of regulatory T cells (Tregs) in allergy protection induced by OIT + FOS.

**Results:**

OIT + FOS decreased acute allergic symptoms and mast cell degranulation upon challenge and prevented the challenge-induced increase in whey-specific IgE as observed in sensitized mice. Early induction of Tregs in the mesenteric lymph nodes (MLN) of OIT + FOS mice coincided with reduced T cell responsiveness in splenocyte cultures. CD25 depletion in OIT + FOS-derived splenocyte suspensions prior to transfer abolished protection against signs of anaphylaxis in recipients. OIT + FOS increased serum galectin-9 levels. No differences in short-chain fatty acid (SCFA) levels in the cecum were observed between the treatment groups. Concisely, FOS supplementation significantly improved OIT in the acute allergic skin response, %Foxp3+ Tregs and %LAP+ Th3 cells in MLN, and serum galectin-9 levels.

**Conclusion:**

FOS supplementation improved the efficacy of OIT in cow’s milk allergic mice. Increased levels of Tregs in the MLN and abolished protection against signs of anaphylaxis upon transfer of CD25-depleted cell fractions, suggest a role for Foxp3+ Tregs in the protective effect of OIT + FOS.

## Introduction

The prevalence of food allergies has been increasing in recent decades, in particular in Western countries. Persistence of food allergies instead of natural outgrowth is observed in patients and is likely to contribute to this increase in the future (1). To date, strict avoidance of the culprit foods and symptomatic treatments are the only options in the management of food allergies. The significant impact of food allergies on health-related quality of life for patients and their families emphasizes the need for safe and efficacious curative treatments (2).

The strategy to induce desensitization and/or oral tolerance to food allergens *via* antigen-specific immunotherapy (AIT) has been studied extensively. Several routes of administration are possible, with the majority of the studies focusing on oral administration. Oral Immunotherapy (OIT) with milk, peanut, and hen’s egg effectively desensitized food allergic patients in randomized controlled clinical trials, measured as the absence of clinical symptoms upon food challenge (3). However, discontinuation of OIT for a period of weeks to months leads to “sustained unresponsiveness” in only a minority of the formerly desensitized patients (3). In addition, safety concerns are relevant, since adverse events ranging from mild to near-fatal reactions have been reported (4). 95% of cow’s milk allergic children subjected to OIT experienced adverse events during treatment, including 25% suffering from severe, frequent, and unpredictable reactions (5). A systematic review and meta-analysis focused on AIT for IgE-mediated food allergies concluded that AIT may be effective in increasing the threshold of reactivity toward allergens, but simultaneously increases the risk of local and systemic adverse events (6). Current limitations regarding safety and long-term protection restrict the use of OIT to treat food allergies in routine clinical practice.

Understanding the mechanism of OIT-induced desensitization and tolerance will contribute to optimizing the therapeutic strategy. A key role has been identified for naturally occurring CD4+ CD25+ Foxp3+ regulatory T cells (Tregs) and inducible type 1 Tregs (Tr1) in securing tolerance toward (food) antigens (7). During immunotherapy, new antigen-specific Tregs are formed under the influence of IL-10 and TGFβ, and they suppress allergen-specific T helper 2 (Th2) and Th1 cells (8). In addition, Tregs control the allergic response by suppressing the antigen-presenting cells responsible for effector T cell induction, shifting the production of antigen-specific IgE to antigen-specific IgG4 and suppressing mast cell and basophil activity (7). Hence, improved Treg responses might be key in successful tolerance induction by OIT.

Nutritional interventions may provide a new window of opportunity to improve the efficacy of OIT for food allergic patients. Dietary non-digestible oligosaccharides (i.e., carbohydrates) mimic the immunomodulatory effects exerted by human milk oligosaccharides (HMOS) in breast-fed infants and have been shown to reduce the risk of developing allergic diseases (9). Non-digestible oligosaccharides show prebiotic activities by stimulating the growth of protective commensal microbes in the gut (10) and are fermented into short-chain fatty acids (SCFA), e.g., butyric acid, by the intestinal bacteria (11). SCFA directly stimulate both immune cells and intestinal epithelial cells (IECs) *via* G-protein coupled receptors and thereby enhance gut integrity (12) and promote oral tolerance (13). In addition to the prebiotic effect, non-digestible oligosaccharides can cross the intestinal epithelial barrier and directly affect immune cells involved in the process of oral tolerance induction (14, 15). The capacity of non-digestible oligosaccharides to induce generic modulation of the immune response (16) and dampen allergic reactions in murine food allergy models (17–19) suggests they may provide a potential benefit in combination with OIT strategies.

With this research, we aimed to assess whether dietary supplementation with non-digestible oligosaccharides supports the efficacy of OIT in a murine cow’s milk allergy (CMA) model, and we aimed to elucidate the potential mechanisms involved. To that end, sensitized female C3H/HeOuJ mice were fed either a control diet or a diet supplemented with plant-derived fructo-oligosaccharides (FOS) and were subjected to OIT for 3 weeks. Subsequently, acute allergic symptoms and mast cell degranulation were measured upon intradermal (i.d.) and intragastric (i.g.) challenges. Blood and organs were collected to measure antigen-specific antibody levels and T- and dendritic cell (DC) populations. Donor spleens derived from sensitized control mice and OIT + FOS mice were used to transfer whole spleen- and CD25-depleted cell fractions to naïve recipient mice to confirm the involvement of Tregs in allergy protection induced by OIT + FOS.

## Materials and Methods

### Diets

A specific mixture of FOS derived from chicory inulin consisted of short-chain FOS [scFOS: oligofructose, Raftilose P95, degree of polymerization (DP) < 6] and long-chain FOS (lcFOS: long-chain inulin, Raftiline HP, average DP 23 or higher, <1% DP < 5) and was provided by Orafti (Wijchen, The Netherlands). FOS were added to the base recipe of the semi-purified cow’s milk protein-free pelleted AIN-93G diet (scFOS/lcFOS ratio 9:1, 1% w/w) at Ssniff Spezialdiäten GmbH (Soest, Germany) (Table 1). The AIN-93G diet without FOS supplementation was used as control diet. Both diets were similar in color and were kept in sealed packages at 4°C prior to use.

**Table 1:** Dietary composition of control diet and FOS supplemented diet.

	Control diet AIN-93G (g/kg)	FOS diet scFOS:lcFOS (9:1, 1%) (g/kg)
**Carbohydrates**		
Cornstarch	397.5	397.5
Dextrinized cornstarch	132.0	132.0
Sucrose	100.0	100.0
**Fibers**		
Arbocel B800	50.0	39.9
Inulin HP (lcFOS) (97%)	0.0	1.03
Raftilose P95 (scFOS) (95%)	0.0	9.47
**Protein**		
Soy protein	200.0	200.0
l-cystine^a^	3.0	3.0
**Fat**		
Soybean oil	70.0	70.0
**Others**		
Mineral mix	35.0	35.0
Vitamin mix	10.0	10.0
Choline bitartrate	2.5	2.5
Tert-butylhydroquinone	0.014	0.014

*^a^0.2% dl-Met and 0.1% l-Cys*.

*FOS, fructo-oligosaccharides; lc, long chain; sc, short chain*.

### Mice

**Figure 1:**
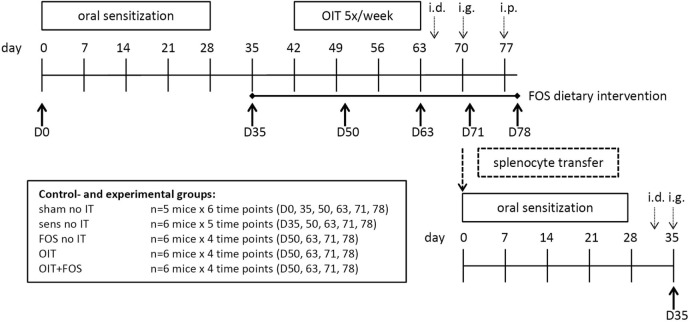
Experimental timeline of the animal experiment. Female C3H/HeOuJ mice were randomly allocated to the control- and experimental groups: sham-sensitized control group (*n* = 5/subgroup); sham no IT, sensitized control group (*n* = 6/subgroup); sens no IT, FOS supplemented group (*n* = 6/subgroup); FOS no IT, OIT group (*n* = 6/subgroup); OIT, and the OIT with FOS supplementation group; OIT + FOS (*n* = 6/subgroup). Mice were i.g. sensitized to the cow’s milk protein whey (20 mg in 0.5 ml PBS) with cholera toxin as an adjuvant (15 µg in 0.5 ml PBS). The FOS-supplemented diet was provided from D35 to the end of the protocol and OIT with 10 mg whey in 0.5 ml PBS was given from D42-D59 (five oral gavages/week for 3 weeks). Acute allergic symptoms were measured upon i.d. challenge at D64 (10 µg whey in 20 µl PBS/ear), mast cell degranulation was measured upon i.g. challenge at D70 (50 mg whey in 0.5 ml PBS), and an i.p. challenge (50 µg whey in 200 µl PBS) was conducted at D77 to stimulate T cell responses prior to organ collection. At 6 time points throughout the animal experiment (D0, D35, D50, D63, D71, and D78), subgroups of mice (*n* = 5–6/group) from each control- and experimental group were killed by cervical dislocation, and blood and organs were collected. Additional groups of donor mice were used in a follow-up experiment to perform the adoptive splenocyte transfer experiment: whole splenocyte suspensions and CD25-depleted fractions collected at D71 from sensitized control mice and OIT + FOS mice (*n* = 8/group) were adoptively transferred to naïve recipient mice (*n* = 6/group). Recipients were sensitized and challenged as described above prior to section at D35. OIT, oral immunotherapy; FOS, fructo-oligosaccharides; i.d., intradermal; i.g., intragastric; i.p., intraperitoneal.

Specific-pathogen-free 6-week-old C3H/HeOuJ female mice were purchased from Charles River Laboratories (Erkrath, Germany). Upon arrival, all mice were randomly allocated to the control and experimental groups: sham-sensitized control group (*n* = 5/subgroup); sham no IT, sensitized control group (*n* = 6/subgroup); sens no IT, FOS supplemented group (*n* = 6/subgroup); FOS no IT, OIT group (*n* = 6/subgroup); OIT, and the OIT with FOS supplementation group; OIT + FOS (*n* = 6/subgroup) (Figure 1). Mice were housed in filter-topped macrolon cages (*n* = 5–6/cage) at the animal facility of Utrecht University, Utrecht, The Netherlands on a 12-h light/dark cycle with unlimited access to food and water. All mice were fed the AIN-93G control diet and were acclimatized for 6 days. Experimental procedures were approved by the Ethical Committee of Animal Research of Utrecht University and complied with the principles of good laboratory animal care following the European Directive for the protection of animals used for scientific purposes.

### Intragastric Sensitization, OIT, and Challenges

The timeline of the animal experiment is depicted in Figure 1. At experimental day (D) 0, 7, 14, 21, and 28, mice were sensitized i.g. to the cow’s milk protein whey (DMV International, Veghel, The Netherlands) dissolved in PBS (20 mg whey in 0.5 ml PBS, Lonza, Verviers, Belgium) using cholera toxin (CT) as an adjuvant (15 µg CT in 0.5 ml PBS, List Biological Laboratories Inc., Campbell, CA, USA). After sensitization (D35), the FOS and OIT + FOS groups received the FOS diet until the end of the protocol. The sham-sensitized control groups, whey-sensitized control groups, and OIT groups were fed the control diet throughout the experiment. Starting at D42, OIT was administered five times per week for three consecutive weeks (i.g. 10 mg whey in 0.5 ml PBS) (D42–D59). On D64, all mice were challenged i.d. in both ear pinnae (10 µg whey in 20 µl PBS/ear) to induce an anaphylactic response. The acute allergic skin response (i.e., ear swelling after i.d. injection), drop in body temperature, and severity of clinical symptoms were measured. Subsequently, mice received an i.g. challenge at D70 (50 mg whey in 0.5 ml PBS) and mast cell-derived mucosal mast cell protease-1 (mMCP-1) was measured in serum samples collected *via* cheek puncture 30 min after challenge. Finally, all mice were challenged intraperitoneally (i.p.) on D77 to stimulate T cell responses prior to organ collection (50 µg whey in 200 µl PBS). At 6 time points throughout the animal experiment (D0, D35, D50, D63, D71, and D78), subgroups of mice (*n* = 5–6/group) from each control- and experimental group were killed by cervical dislocation, and blood and organs were collected (Figure 1). Additional groups of donor mice were used in a follow-up experiment to perform the adoptive splenocyte transfer experiment and histological analyses in colon tissue.

### Acute Allergic Skin Response, Body Temperature and Anaphylaxis Symptom Scores upon i.d. Challenge

The magnitude of the acute allergic skin response after i.d. injection of the allergen was measured as Δ ear swelling by subtracting the mean basal ear thickness from the mean ear thickness 1 h post-challenge (in duplicate in both ears). Ear thickness in micrometers was measured using a digital micrometer (Mitutoyo, Veenendaal, The Netherlands). After blinding the cages, all mice were anesthetized using inhalation of isoflurane to perform the i.d. injection and both ear measurements [*n* = 10–12/group, data represent the mean of two subgroups: mice scheduled for section at D71 and D78 (Figure 1)]. Body temperature was measured 45 min after i.d. challenge to monitor the anaphylaxis-associated drop in temperature using a rectal thermometer and severity of anaphylaxis symptoms was scored according to the method described by Li et al. (20).

### Adoptive Splenocyte Transfer

At D71, pooled spleen suspensions derived from sensitized control- and OIT + FOS mice (*n* = 8/group) from a follow-up experiment were adoptively transferred to naïve specific-pathogen-free 6-week-old C3H/HeOuJ female mice (Charles River Laboratories, *n* = 6/group), which were fed the control diet and were housed under similar conditions as described earlier. After homogenization and red blood cell lysis, whole spleen suspensions and CD25-depleted fractions were intravenously (i.v.) injected into the tail vein of recipient mice (1 × 10^6^ cells in 100 µl PBS) prior to sensitization and i.d. and i.g. challenges as described in previous sections. CD25-depleted CD4+ fractions were obtained using a CD4+ CD25+ purification kit according to the manufacturer’s instructions (Miltenyi Biotec, Leiden, The Netherlands). Flow cytometric analysis of CD25-depleted fractions showed that <1% of the cells were positive for Foxp3. In addition, whole spleen suspensions showed on average 14% Foxp3 positivity. At D35 after transfer, recipient mice were killed by cervical dislocation 30 min after i.g. challenge, and blood and spleens were collected for further analysis.

### Serum Levels of Whey-Specific Antibodies, mMCP-1 and Galectin-9

Blood was collected *via* cheek puncture at D0, D35, D50, D63, D71, and D78 prior to sectioning of the mice and from one corresponding subgroup (*n* = 10–12 samples/group) and at D35 in case of the recipient mice (*n* = 6 samples/group). Blood samples were centrifuged (10,000 rpm for 10 min) and serum was stored at −20°C until analysis of mMCP-1, whey-specific antibodies, and galectin-9 by means of ELISA. Determination of whey-specific antibodies was performed as described previously (21). Concentrations of mMCP-1 in serum collected 30 min after i.g. challenge [D70; *n* = 10–12 samples/group, data represent the mean of two subgroups: mice scheduled for section at D71 and D78 (Figure 1) and D35 in recipients; *n* = 6/group] were measured by using a mMCP-1 Sandwich ELISA Kit (Mouse MCPT-1 ELISA Ready-SET-Go kit, eBioscience, Breda, The Netherlands) according to the manufacturer’s instructions. Serum collected from mice killed at D50, D63, and D71 was used to measure galectin-9 concentrations. Overnight incubation (4°C) with 100 µl of coating antibody in coating buffer (0.75 µg/ml, mouse galectin-9 affinity purified polyclonal goat IgG antibody, R&D Systems, Oxon, UK) in 96-wells high-binding plates (Corning Incorporated, Corning, NY, USA) was followed by a washing step. The wells were blocked for 1 h (RT) with 200 µl blocking buffer (PBS with 1% BSA) and washed prior to 2 h (RT) incubation of serum samples diluted (100×) in dilution buffer (PBS with 1% BSA and 0.05% Tween20) and a standard curve with recombinant mouse galectin-9 (serial 1:1 dilution starting with 500,000 pg/ml, R&D Systems). After washing, the plates were incubated with 100 µl capture antibody (0.75 µg/ml, mouse galectin-9 biotinylated affinity purified goat IgG antibody, R&D Systems) for 1 h at RT. Afterward, the plates were washed and incubated with 100 µl Strep-HRP (Sanquin, Amsterdam, The Netherlands) for 1 h at RT in the dark. The color reaction was initiated by adding 100 µl 3,3′,5,5′-tetramethylbenzidine (1-Step Ultra TMB, Thermo Fisher Scientific, Waltham, MA, USA), and the reaction was stopped with 4 M H_2_SO_4_ (50 µl). Optical density was measured with a Benchmark microplate reader (BioRad, Hercules, CA, USA) at a wavelength of 450 nm.

### Spleen, Mesenteric Lymph Nodes (MLN), and Lamina Propria (LP) Cell Isolation

Spleen and MLN were collected (*n* = 5–6/group) at D0, D35, D50, D63, D71, and D78 and homogenized using a syringe and a 70-µm cell strainer. Red blood cells were lysed in the splenocyte suspensions using lysis buffer (8.3 g NH_4_Cl, 1 g KHC_3_O, and 37.2 mg EDTA dissolved in 1 L demi water, filter sterilized). Cell suspensions were either dissolved in RPMI 1640 medium (Lonza) supplemented with 10% fetal bovine serum (FBS) and penicillin (pen, 100 U/ml)/streptomycin (strep, 100 µg/ml, Sigma-Aldrich Chemicals, Zwijndrecht, The Netherlands) and β-mercaptoethanol (20 µM) prior to *ex vivo* antigen-specific stimulation assays or dissolved in FACS buffer (PBS with 1% BSA) prior to flow cytometry stainings.

At D63 and D71, small intestine LP tissue (*n* = 4/group) was collected to isolate lymphocytes as follows: fat and Peyer’s patches were removed from small intestine tissue and after washing in Hank’s Balanced Salt Solution (HBSS, Invitrogen, Life Technologies, Carlsbad, CA, USA) with 15 mM Hepes (Gibco, Life Technologies) at pH 7.2, longitudinally opened tissue was cut into small fragments (0.5 cm). After washing in HBSS/Hepes, tissue samples were incubated in HBSS/Hepes buffer supplemented with 10% FBS, pen/strep, 5 mM EDTA, and 1 mM dithiothreitol (DTT) for 20 min at 37°C (2×). Subsequently, tissue samples were washed in RPMI/FBS/DTT to remove EDTA followed by incubation in RPMI/FBS/DTT with collagenase type D (1 mg/ml, Roche Diagnostics Inc., Almere, The Netherlands) and DNAse (20 µg/ml, Sigma) for 45 min on a plate shaker at 37°C (2×). After digestion, remaining tissue fragments were re-suspended using a syringe (10 ml) and a needle (18 G) and suspensions were filtered with a 100-µm cell strainer afterward. LP-derived cell suspensions were washed in HBSS/Hepes and purified using a percoll (GE Healthcare, Uppsala, Sweden) 40–80% mediated separation after centrifugation. Purified cell fractions were washed to remove traces of percoll and cells were taken up in FACS buffer prior to flow cytometry stainings.

### *Ex Vivo* Antigen-Specific Stimulation of Splenocytes for Cytokine Measurements

8 × 10^5^ cells/well in 200 µl culture medium (RPMI 1640, 10% FBS, pen/strep, β-mercaptoethanol) in 96-wells U-bottom plates (Greiner, Frickenhausen, Germany) were stimulated with either culture medium, anti-CD3 (1 µg/ml, eBioscience) or anti-CD3/anti-CD28 (10 µg/ml anti-CD3 and 1 µg/ml anti-CD28, eBioscience, transfer experiment) or whey (50 µg/ml). Polyclonal stimulation (48 h) and whey stimulation (96 h) were conducted at 37°C and 5% CO_2_. Culture supernatant was collected and stored at −20°C until measurements of IL-5, IL-10, IL-13, and IFNγ production by means of ELISA according to the protocol described earlier for galectin-9. Purified rat anti-mouse coating antibodies (1 µg/ml for IL-5 and IFNγ and 2 µg/ml for IL-10 and IL-13), recombinant mouse cytokines for the standard curve, and biotinylated detection antibodies (1 µg/ml for IL-5, IL-10 and IFNγ and 400 ng/ml for IL-13) were purchased at BD Biosciences.

### Flow Cytometry

To increase the expression of latency-associated peptide (LAP) on the surface of MLN-derived lymphocytes, cells were incubated in culture medium (RPMI 1640, 10% FBS, pen/strep) and received polyclonal stimulation with anti-CD3/anti-CD28 (10 µg/ml anti-CD3 and 1 µg/ml anti-CD28, eBioscience) for 24 h at 37°C and 5% CO_2_ prior to staining. Otherwise spleen, MLN, and LP-derived cell suspensions in FACS buffer were plated in 96-wells U-bottom Falcon plates (BD Biosciences, 1–0.5 × 10^6^ cells/well). The cells were incubated with anti-mouse CD16/CD32 (mouse Fc Block, BD Biosciences) in FACS buffer to block non-specific binding sites (15 min, on ice). Subsequently, cells were stained extracellularly with the following fluorescent antibodies (all purchased at eBioscience unless stated otherwise) in FACS buffer for 30 min on ice in the dark: anti-CD4-PerCpCy5.5 (1:100, clone RM4-5), anti-CD69-APC (1:100, clone H1.2F3), anti-CXCR3-PE (1:50, clone CXCR3-173), anti-T1St2-FITC (1:50, clone DJ8, mdbioproducts, St. Paul, MN, USA), anti-CD25-AlexaFluor 488 (1:100, clone PC61.5), anti-F4/80-APC-eFluor 780 (1:100, clone BM8), anti-CD103-APC (1:100, clone 2E7), anti-CD11b-PE (1:50, clone M1/70), anti-CD11c-PerCpCy5.5 (1:50, clone N418), anti-MHCII-FITC (1:100, clone NIMR-4), anti-CD45-PE-Cy7 (1:100, clone 30-F11), anti-CD4-FITC (1:100, clone GK1.5), and anti-LAP-PerCP-eFluor 710 (1:50, clone TW7-16B4). Cells stained for extracellular markers were fixed using 1% IC fixation buffer (eBioscience), and cells receiving additional intracellular staining for the transcription factor Foxp3 were fixed and permeabilized using the Foxp3 staining buffer set purchased at eBioscience according to the manufacturer’s instructions. Afterward, cells were incubated for 30 min on ice in the dark with anti-Foxp3-APC (1:50, clone FJK-16s) in permeabilization buffer. Live cells were distinguished from dead cells using Fixable Viability Dye eFluor 780 (FVD, 1:2000, eBioscience) and single cells were separated from aggregated cells based on forward/sideward scatter properties. Isotype controls were used for each antibody and cutoff gates for positivity were established using the fluorescence-minus-one technique. Fluorescence was measured on the FACS Canto II (BD Biosciences) and analyzed with Flowlogic software (Inivai Technologies, Mentone, Australia).

### Histological Staining for Mast Cells and Foxp3+ Tregs in Colon Tissue

The colon was dissected (*n* = 3–6/group), opened longitudinally, washed in PBS, and Swiss roles were prepared by rolling the tissue from distal to proximal end with the mucosal side down. Tissue roles were fixed in formalin (10% v/v) and embedded in paraffin (Leica IG1150c, Leica Microsystems, Rijswijk, The Netherlands). Tissue sections (5 µm) were cut with a microtome (Leica Microsystems) and mounted on slides prior to deparaffinization and hydration. To stain mast cells in the tissue sections, May-Grunwald and Giemsa solutions were used according to the manufacturer’s instructions (Giemsa Stain, Abcam, Cambridge, UK). Intracellular Foxp3 expression was stained as described previously (22). After dewaxing, the sections were boiled in sodium citrate buffer (0.01 M) for 15 min. Then the sections were incubated with 0.2% Tween20 in PBS for 20 min. After blocking with 5% rabbit serum (Dako, Heverlee, Belgium) in PBS with 1% BSA (PBS/BSA) for 30 min, the sections were incubated overnight (4°C) with rat anti-mouse Foxp3 purified antibody (10 µg/ml, eBioscience). Afterward, sections were incubated with 3% H_2_O_2_ in PBS for 30 min. Detection of the primary antibody was conducted with a biotinylated rabbit anti-rat IgG (2.5 µg/ml in PBS/BSA, Jackson ImmunoResearch, West Grove, PA, USA) for 1 h (RT). Then sections were incubated with avidin biotin complex (ABC HRP kit, Vector Laboratories, Peterborough, UK) in PBS/BSA for 1 h. The color reaction was developed with 3,3′-diaminobenzidine (Sigma) and counterstaining was performed with hematoxylin. After dehydration, the sections were covered with Pertex mounting medium (Histolab, Göteborg, Sweden) and cover glass. Stained cells were counted per 100 intact cripts in each colon section.

### Foxp3 and IL-10 mRNA Expression in Colon Tissue

One centimetre of the proximal colon was dissected and stored in RNAlater (Sigma) at −80°C until further processing (*n* = 5–6/group). After homogenization, RNA extraction was conducted using the Qiagen RNeasy isolation kit and the RNAse-free DNAse Set (Qiagen GmbH, Hilden, Germany) and cDNA was synthesized using the iScript™ cDNA synthesis kit (Biorad) according to the manufacturer’s instructions. RT^2^ qPCR Primer Assays to measure pPIP5k1 (housekeeping gene), Foxp3 and IL-10 were purchased at SA Biosciences (Qiagen, German Town, MD, USA) and quantitative real-time PCR was performed on a CFX96 real-time PCR detection system (Biorad) using iQ SYBR green supermix as described previously (19). Foxp3 and IL-10 mRNA expression data were normalized to pPIPk1 and depicted as the fold change in expression compared to the sham-sensitized control group.

### SCFA Analysis in Cecum Content

Short-chain fatty acid analysis was conducted as described elsewhere (23). Briefly, cecum content was collected (*n* = 5–6/group) and frozen at −80°C until further analysis. Samples were defrosted, homogenized by vortexing, and diluted in ice cold PBS (1:10). After centrifugation (13,000 rpm for 10 min), the supernatant was analyzed using a Shimadzu GC2010 gas chromatograph (Shimadzu Corporation, Kyoto, Japan) and acetic, propionic, butyric, iso-butyric, valeric, and iso-valeric acid concentrations were quantitated (based on 2-ethylbutyric acid internal standard).

### Data Analysis and Statistics

Data are depicted as mean ± SEM and were statistically analyzed with GraphPad Prism software version 6.00 (GraphPad software, La Jolla, CA, USA) using one-way ANOVA and Bonferroni’s *post hoc* test to compare pre-selected combinations. Calculated *p*-values were adjusted for the total number of comparisons made and were considered statistically significant when *p* < 0.05. In addition, whey-specific antibody data were log transformed prior to testing. The anaphylaxis symptom scores were analyzed using Kruskal–Wallis test for non-parametric data with Dunn’s *post hoc* test to compare pre-selected combinations.

## Results

### Allergic Symptoms

**Figure 2:**
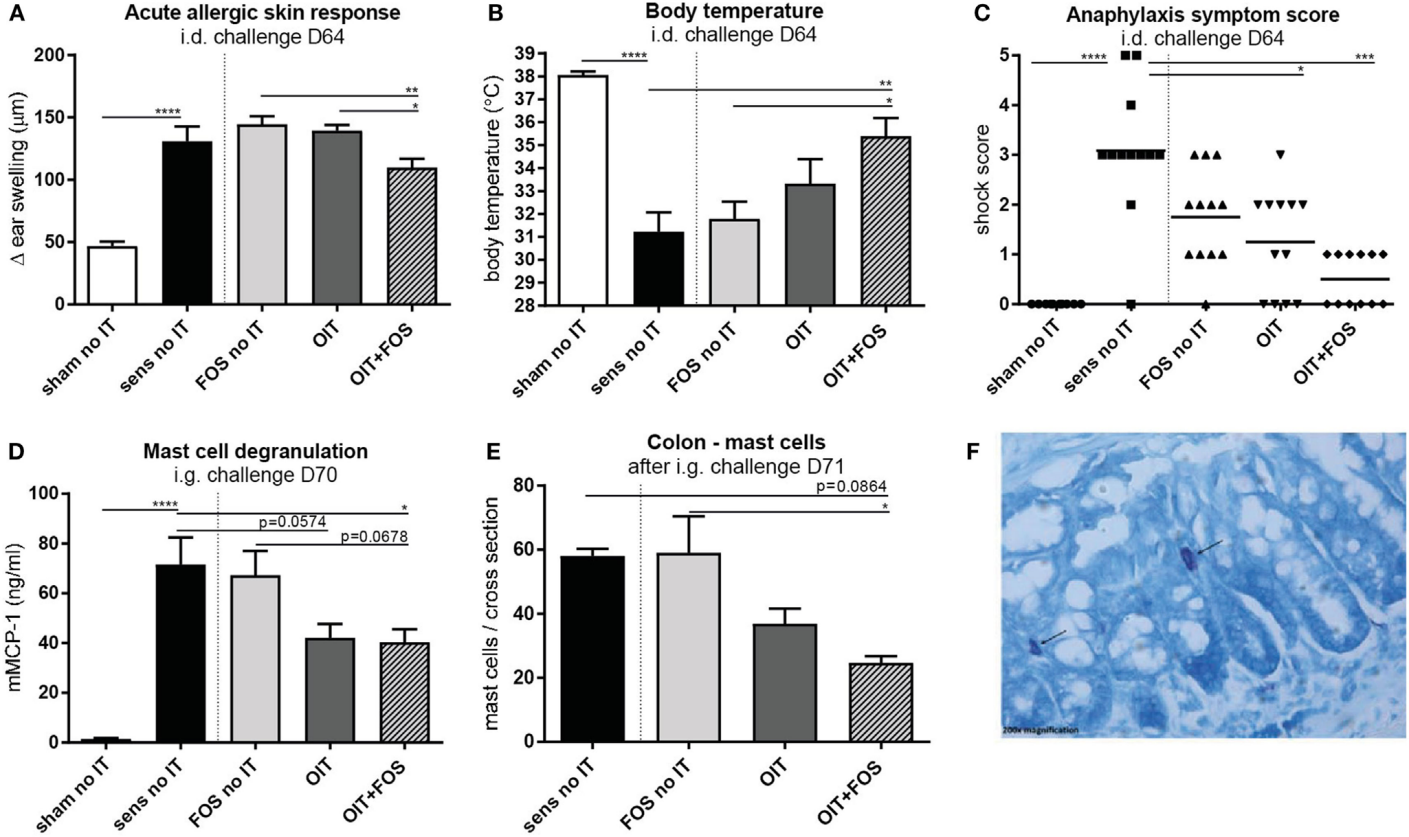
Acute allergic symptoms and mast cell degranulation upon challenge. **(A)** Acute allergic skin response measured as Δ ear swelling 1 h after i.d. injection at D64. **(B)** Body temperature levels and **(C)** anaphylaxis symptom scores during anaphylaxis measured 45 min after i.d. challenge at D64. **(D)** Serum mMCP-1 concentrations 30 min after i.g. challenge at D70. **(E)** Mast cell numbers in colon Swiss role cross sections after May-Grunwald/Giemsa staining (total number in one cross section). **(F)** Representative slide with stained mast cell (OIT + FOS). Data are represented as mean ± SEM *n* = 10–12/group in **(A–D)** and *n* = 3–6/group in **(E)**. Statistical analysis was performed using one-way ANOVA and Bonferroni’s *post hoc* test and anaphylaxis symptom scores were analyzed using Kruskal–Wallis test for non-parametric data with Dunn’s *post hoc* test. *p < 0.05, **p < 0.01, ***p < 0.001, ****p < 0.0001. Sens, sensitization; OIT, oral immunotherapy; FOS, fructo-oligosaccharides; no IT, no immunotherapy; i.d., intradermal; i.g., intragastric; mMCP-1, mucosal mast cell protease-1.

Sensitization to the cow’s milk protein whey increased the acute allergic skin response measured as ear swelling (*p* < 0.0001) and caused severe anaphylaxis (*p* < 0.0001) with a characteristic drop in body temperature (*p* < 0.0001) upon i.d. challenge (D64) compared to sham-sensitization (Figures 2A–C). An increased serum mMCP-1 concentration, indicative for mast cell degranulation, was observed upon i.g. challenge (D70) in the sensitized control animals compared to sham-sensitized control animals (*p* < 0.0001) (Figure 2D). OIT + FOS induced protection against these allergic symptoms: the acute allergic skin response was reduced after OIT + FOS compared to FOS (*p* = 0.0048) or OIT alone (*p* = 0.023) (Figure 2A), indicating the improved efficacy of the combination strategy. OIT + FOS protected against the anaphylaxis-associated drop in body temperature compared to sensitized control mice (*p* = 0.0045) and FOS no IT (*p* = 0.0123) (Figure 2B) and both OIT and OIT + FOS reduced the severity of anaphylaxis symptoms (Figure 2C) compared to the sensitized control (*p* = 0.0322 and *p* = 0.0002, respectively). After i.g. challenge (D70), OIT + FOS reduced mast cell degranulation (mMCP-1) compared to the sensitized control (*p* = 0.0321) (Figure 2D). The reduction in mMCP-1 coincided with reduced local mast cell numbers in colon tissue of OIT + FOS mice, but only compared to FOS supplementation alone (FOS no IT, *p* = 0.0227) (Figures 2E,F).

### Whey-Specific Antibody Levels in Serum

**Figure 3:**
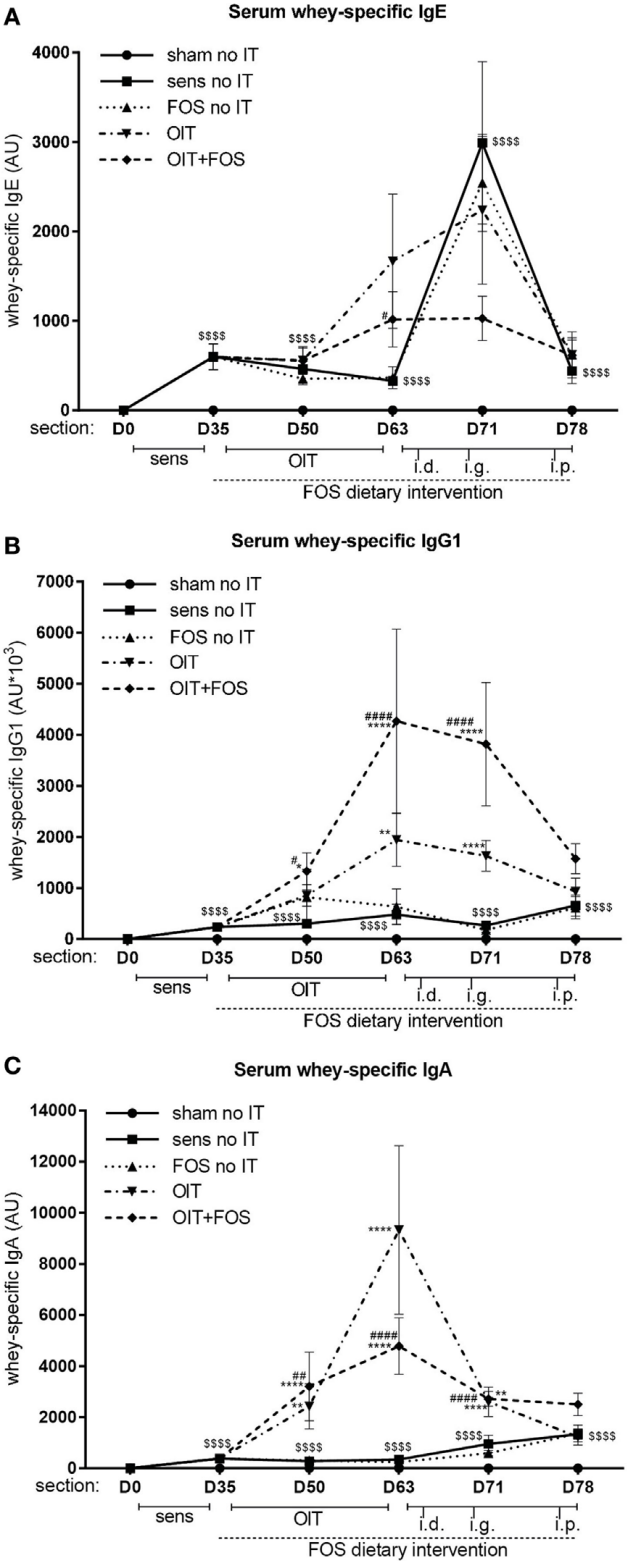
Whey-specific IgE, IgG1, and IgA levels in serum. **(A)** Whey-specific IgE levels, **(B)** Whey-specific IgG1 levels, and **(C)** Whey-specific IgA levels in serum measured by means of ELISA. Data are log transformed and represented as mean ± SEM *n* = 10–12/group/time point and *n* = 5–6/group at D78 in **(A–C)**. Statistical analysis was performed using one-way ANOVA and Bonferroni’s *post hoc* test per individual time point. ^$$$$^*p* < 0.0001 for whey-sensitized control group compared to sham-sensitized control group. *p < 0.05, **p < 0.01, ***p < 0.001, ****p < 0.0001 compared to sensitized control group. ^#^*p* < 0.05, ^##^*p* < 0.01, ^###^*p* < 0.001, ^####^*p* < 0.0001 compared to FOS no IT group. Sens, sensitization; OIT, oral immunotherapy; FOS, fructo-oligosaccharides; no IT, no immunotherapy; i.d., intradermal; i.g., intragastric; i.p., intraperitoneal; AU, arbitrary units.

Oral sensitization to whey increased the level of whey-specific IgE, IgG1, and IgA in serum compared to sham-sensitized mice (*p* < 0.0001) (D35, D50, D63, D71, and D78) (Figures 3A–C). As observed after immunotherapy (D63), OIT increased whey-specific IgG1 and IgA levels compared to sensitized controls (*p* = 0.0026 for IgG1 and *p* < 0.0001 for IgA) and a similar pattern was observed for IgE (Figures 3A–C). FOS supplementation did not affect the OIT-induced rise in whey-specific antibodies, since no differences were observed between OIT and OIT + FOS. Whey-specific IgG1 and IgA levels decreased despite a series of challenges (D63–D78) in the OIT and OIT + FOS groups (Figures 3B,C). Interestingly, OIT + FOS protected against the rise in whey-specific IgE (D71) observed after i.d. and i.g. challenge, since levels were comparable to the time point preceding the challenges (D63) (Figure 3A).

### T Helper Cell Subsets in Lymphoid Organs

**Figure 4:**
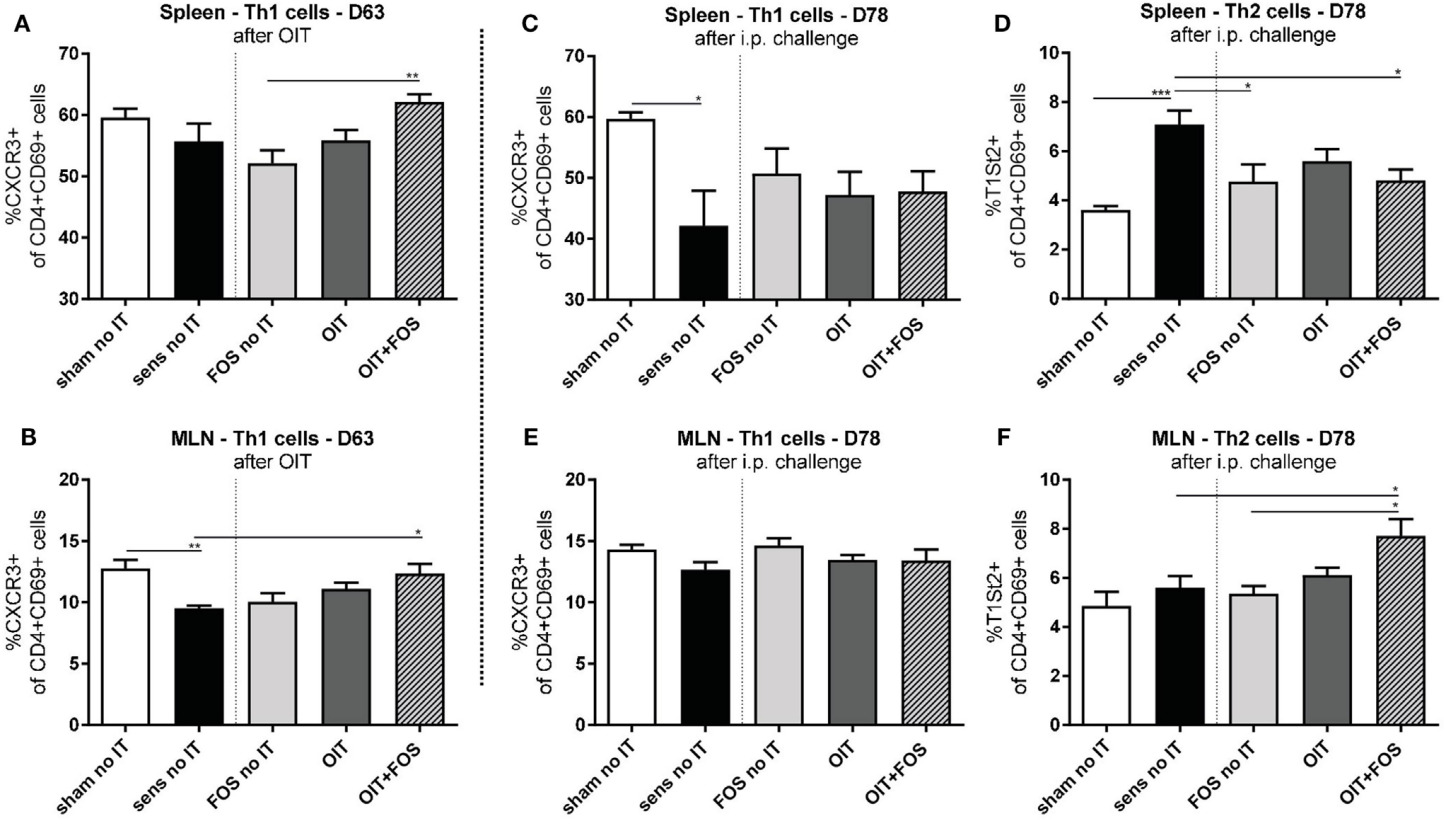
Flow cytometric analysis of T helper cell populations in spleen and MLN. **(A)** Percentage of activated Th1 cells (CXCR3+ of CD4+ CD69+ cells) in spleen and **(B)** in MLN after immunotherapy (D63). **(C)** Percentage of activated Th1 cells and **(D)** Th2 cells (T1St2+ of CD4+ CD69+ cells) in spleen after i.p. challenge (D78). **(E)** Percentage of activated Th1 cells and **(F)** Th2 cells in MLN after i.p. challenge (D78). Data are represented as mean ± SEM *n* = 5–6/group in **(A–F)**. Statistical analysis was performed using one-way ANOVA and Bonferroni’s *post hoc* test. *p < 0.05, **p < 0.01, ***p < 0.001, ****p < 0.0001. Sens, sensitization; OIT, oral immunotherapy; FOS, fructo-oligosaccharides; no IT, no immunotherapy; i.p., intraperitoneal; Th, T helper; MLN, mesenteric lymph nodes.

No differences in Th1 and Th2 cell percentages were shown between OIT and OIT + FOS animals, however, differences were observed at D63 (after OIT) and D78 (after i.p. challenge) between OIT + FOS mice and sensitized controls (Figure 4). At D63, an increase in activated Th1 cells (CXCR3+ of CD4+ CD69+) was observed in the OIT + FOS group compared to the sensitized control group in the MLN (*p* = 0.0159) (Figure 4B) and compared to the FOS no IT group in the spleen (*p* = 0.0096) (Figure 4A). At D78, activated Th2 cells (T1St2+ of CD4+ CD69+) were increased in the sensitized control animals compared to sham-sensitized control animals (*p* = 0.0009) and both FOS (*p* = 0.0259) and OIT + FOS (*p* = 0.0419) reduced the percentage of Th2 cells in spleen (Figure 4D). No differences were observed in the MLN at D78, except the elevated percentage of activated Th2 cells in the OIT + FOS group compared to the sensitized control (*p* = 0.0297) and FOS no IT (*p* = 0.0121) groups (Figures 4E,F).

### Induction of Tolerance-Associated Cell Types

**Figure 5:**
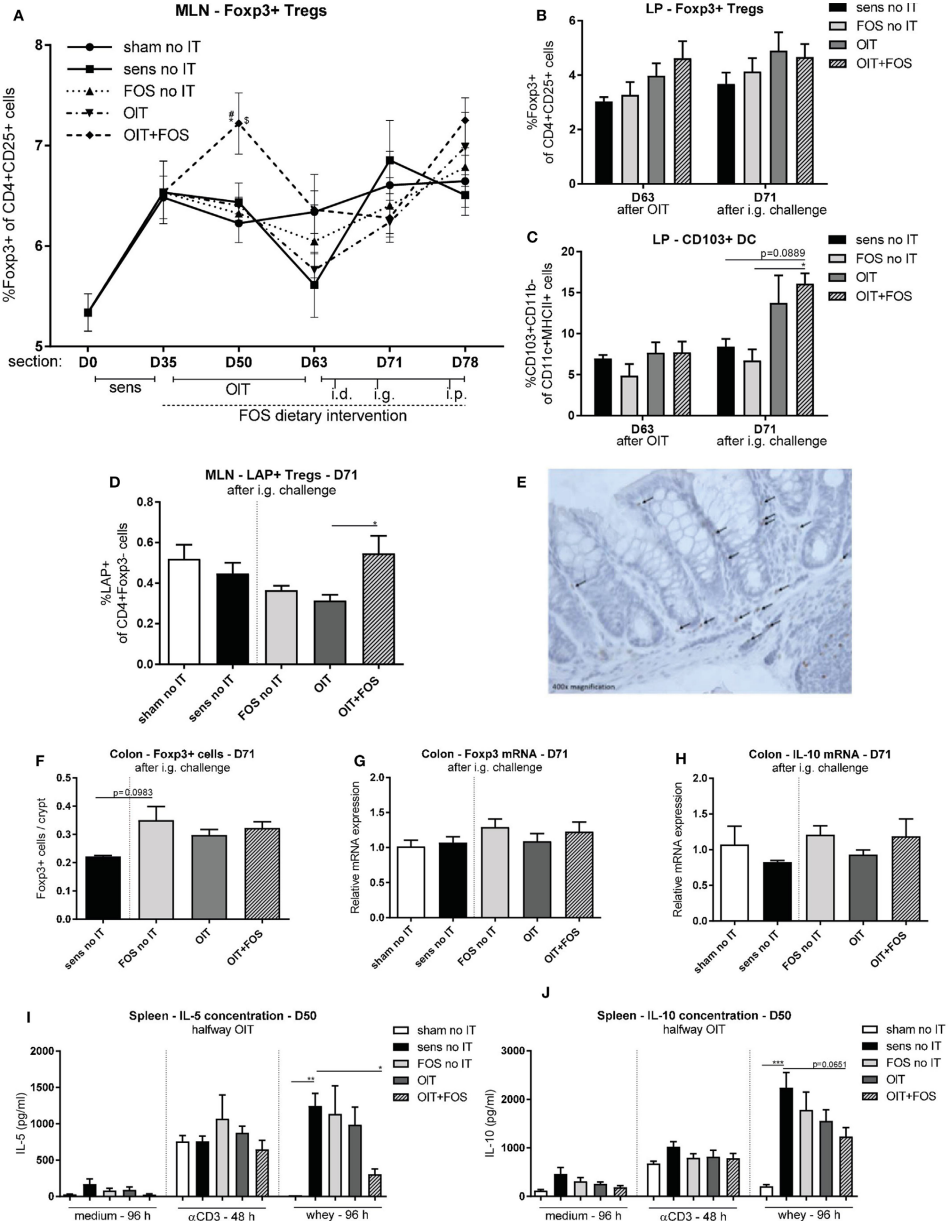
Treg analysis with flow cytometry, immunohistochemistry, and qPCR. **(A)** Percentage of CD4+ CD25+ Foxp3+ cells in MLN measured with flow cytometry. **(B)** Percentage of CD4+ CD25+ Foxp3+ cells in LP measured with flow cytometry after immunotherapy (D63) and after i.g. challenge (D71). **(C)** Percentage of CD103+ DCs (CD103+ CD11b− of CD11c+ MHCII+ cells) in LP measured with flow cytometry after immunotherapy (D63) and after i.g. challenge (D71). **(D)** Percentage of LAP+ Th3 cells (LAP+ of CD4+ Foxp3− cells) in MLN measured with flow cytometry after i.g. challenge (D71). **(E)** Representative slide with Foxp3+ cells (OIT). **(F)** Foxp3+ cells in colon Swiss role cross sections stained with immunohistochemistry and counted in at least 100 intact crypts. **(G)** Relative mRNA expression of Foxp3 and **(H)** IL-10 in colon tissue measured with qPCR. **(I)** IL-5 concentrations and **(J)** IL-10 concentrations in supernatant after *ex vivo* stimulation of splenocytes with medium, anti-CD3, and whey halfway immunotherapy (D50). Data are represented as mean ± SEM *n* = 5–6/group in **(A,D,G–J)**, *n* = 4/group in **(B,C)** and *n* = 3–6/group in **(F)**. Statistical analysis was performed using one-way ANOVA and Bonferroni’s *post hoc* test. *p < 0.05, **p < 0.01, ***p < 0.001, ****p < 0.0001 [**(A)**: *compared to sens no IT, ^#^compared to FOS no IT, ^$^compared to OIT]. Sens, sensitization; OIT, oral immunotherapy; FOS, fructo-oligosaccharides; no IT, no immunotherapy; i.d., intradermal; i.g., intragastric; i.p., intraperitoneal; Treg, regulatory T cell; Th, T helper; DC, dendritic cell; MLN, mesenteric lymph nodes; LP, lamina propria; LAP, latency-associated peptide.

As shown in Figure 5A, an early increase in the percentage of CD4+ CD25+ Foxp3+ Tregs was observed halfway immunotherapy (D50) in the MLN of the OIT + FOS mice compared to OIT (*p* = 0.0457). The higher percentage of Foxp3+ Tregs in MLN coincided with reduced IL-5 concentrations upon antigen-specific stimulation of splenocytes derived from OIT + FOS mice (D50) compared to the sensitized control (*p* = 0.0365) (Figure 5I). A similar pattern was found for IL-10 (*p* = 0.0651; not significant) (Figure 5J). No differences in cytokine concentrations were observed after medium and anti-CD3 stimulation of splenocytes (Figures 5I,J). At D63, the percentage of Foxp3+ Tregs in LP-derived lymphocyte fractions suggests an increase in the OIT + FOS mice compared to sensitized control mice, however, these results are not statistically significant (Figure 5B). A tendency toward increased percentages of CD103+ CD11b− DCs in LP was observed in the OIT + FOS group compared to the sensitized control (*p* = 0.0889; not significant) after challenge (D71), but not after immunotherapy (D63) (Figure 5C). The percentage of LAP+ Tregs was increased in the OIT + FOS group compared to OIT at D71 in MLN (*p* = 0.0327) (Figure 5D). Levels of Foxp3+ cells and Foxp3 and IL-10 mRNA expression in colon tissue were not statistically different between the groups at D71 (Figures 5E–H). A trend (*p* = 0.0983) toward an increase in the number of Foxp3+ cells was observed in colon samples of FOS-treated animals compared to sensitized control animals (Figure 5F).

### Transfer of Protection against Allergic Symptoms

**Figure 6:**
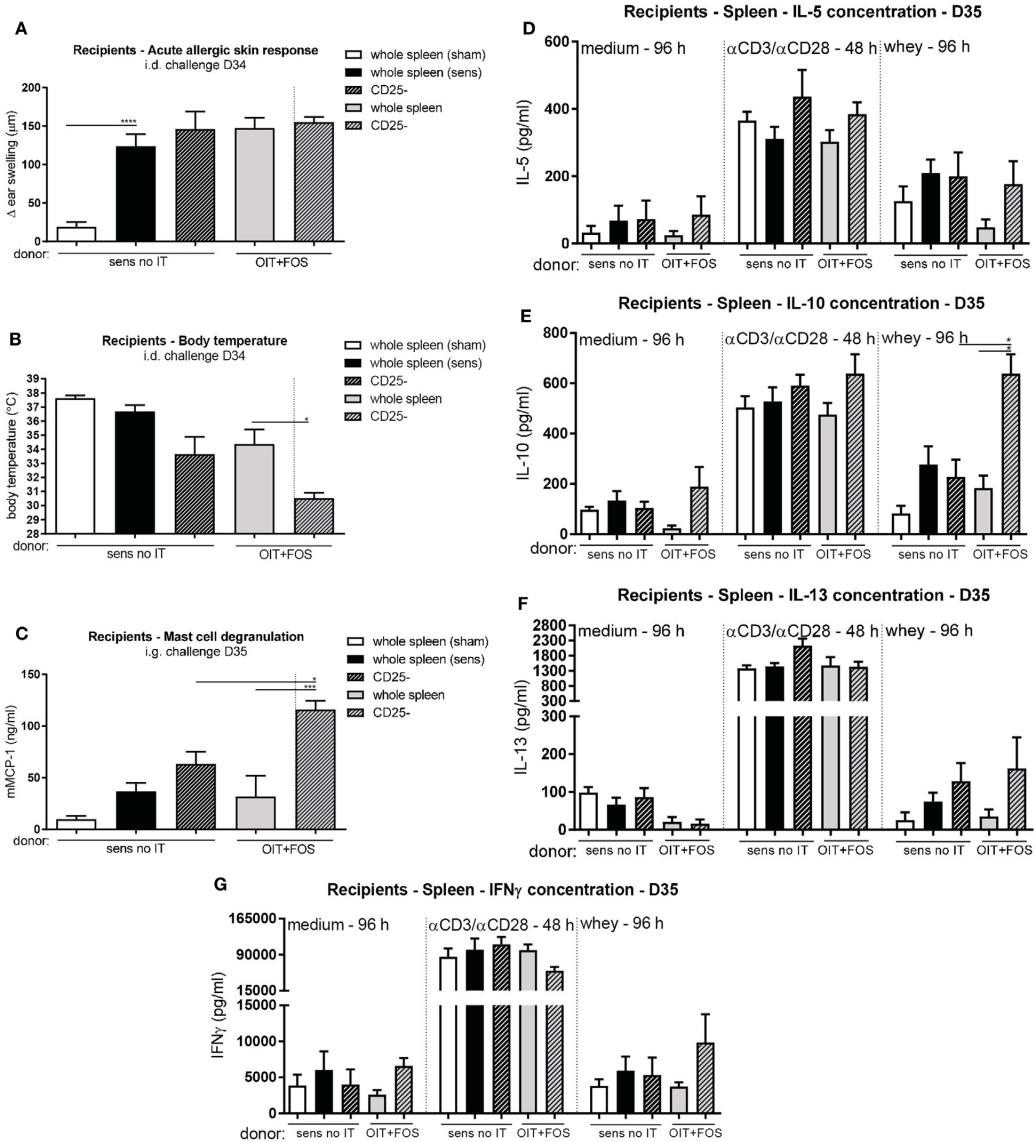
Adoptive cell transfer of whole spleen- and CD25-depleted pooled splenocyte suspensions derived from sensitized control mice and OIT + FOS mice. **(A)** Acute allergic skin response measured as Δ ear swelling 1 h after i.d. injection at D34 in the recipients. **(B)** Body temperature levels during anaphylaxis measured 45 min after i.d. challenge at D34 in the recipients. **(C)** Serum mMCP-1 concentrations 30 min after i.g. challenge at D35 in the recipients. **(D)** IL-5 concentrations, **(E)** IL-10 concentrations, **(F)** IL-13 concentrations, and **(G)** IFNγ concentrations in supernatant after *ex vivo* stimulation of recipients-derived splenocytes with medium, anti-CD3/anti-CD28, and whey. Data are represented as mean ± SEM *n* = 6/group in **(A–G)**. Statistical analysis was performed using one-way ANOVA and Bonferroni’s *post hoc* test. *p < 0.05, **p < 0.01, ***p < 0.001, ****p < 0.0001. Sens, sensitization; OIT, oral immunotherapy; FOS, fructo-oligosaccharides; no IT, no immunotherapy; mMCP-1, mucosal mast cell protease-1; i.d., intradermal; i.g., intragastric.

To confirm the involvement of the induced Foxp3+ Tregs in protection against allergic symptoms, pooled whole spleen suspensions and CD25-depleted fractions derived from sensitized control mice and OIT + FOS mice were adoptively transferred to naïve recipients. In contrast to body temperature (Figure 6B), challenge-induced acute allergic skin responses were significantly increased in the whey-sensitized recipient control mice compared to the sham-sensitized recipient control mice (*p* < 0.0001), indicating that cell transfer was not responsible for the allergen-induced ear swelling response (Figure 6A). OIT + FOS splenocyte transfer did not protect against the increased ear swelling response (Figure 6A). CD25 depletion induced a more severe drop in body temperature in the OIT + FOS recipients compared to the recipients of the whole spleen suspension (*p* = 0.0426) (Figure 6B). Similarly, challenge-induced mast cell activation was increased in mice which received the CD25-depleted fraction compared to the recipients of the OIT + FOS whole spleen suspension (*p* = 0.0003) (Figure 6C). In addition, CD25 depletion increased the IL-10 concentration in whey-stimulated splenocyte cultures derived from OIT + FOS recipients (*p* = 0.0125) (Figure 6E). A similar pattern was observed for IL-5, IL-13, and IFNγ; however, differences were not significant (Figures 6D,F,G). No differences in cytokine concentrations were observed after medium and anti-CD3/anti-CD28 stimulation (Figures 6D–G).

### Direct and Indirect Modulation of the Intestinal Environment by OIT + FOS

**Figure 7:**
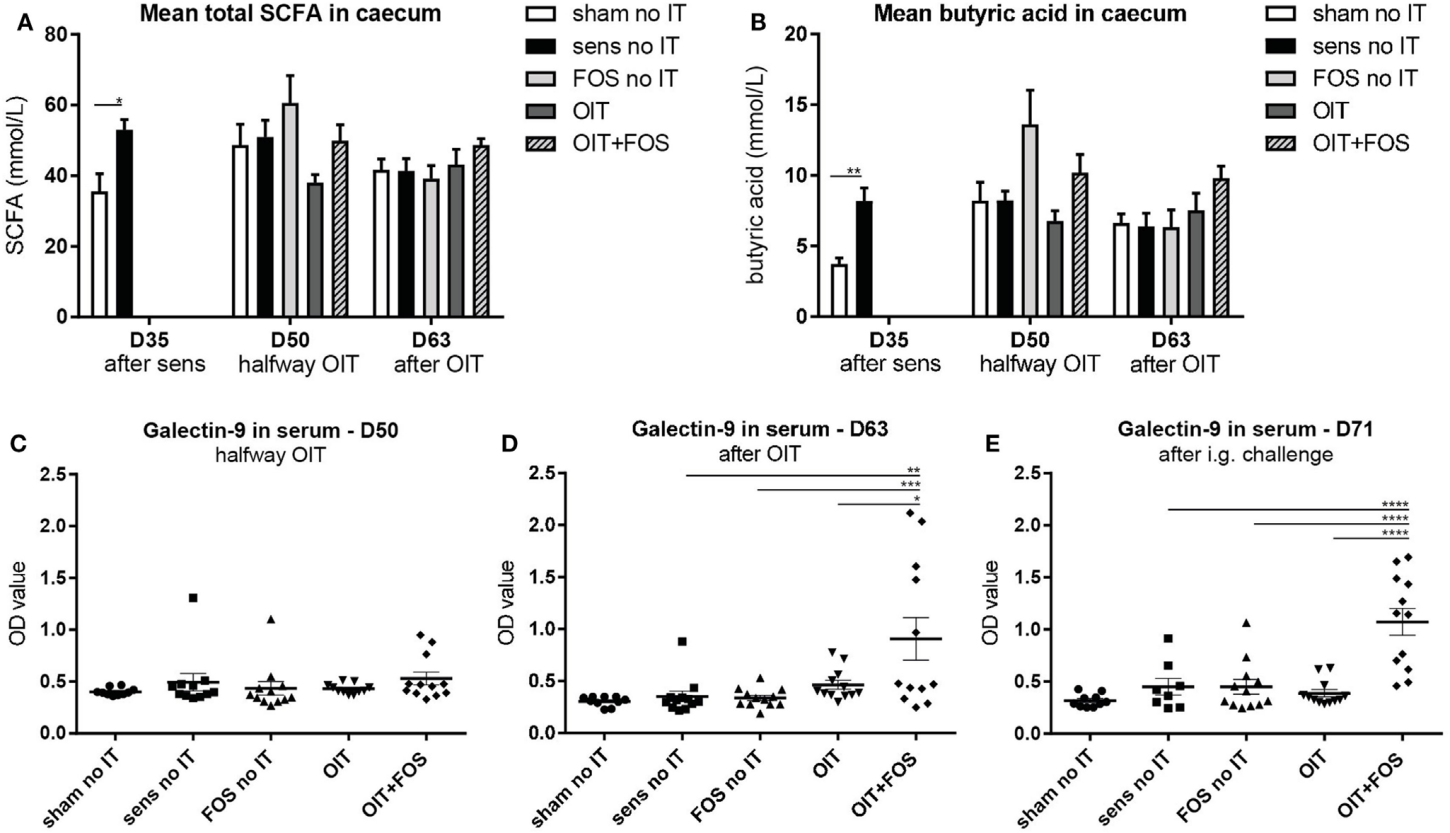
SCFA analysis in cecum content and serum galectin-9 levels. **(A)** Mean total SCFA concentrations and **(B)** mean butyric acid concentrations in homogenized cecum content supernatant collected after sensitization (D35), halfway immunotherapy (D50) and after immunotherapy (D63). **(C)** Serum galectin-9 levels halfway immunotherapy (D50), **(D)** after immunotherapy (D63) and **(E)** after i.g. challenge (D71). Data are represented as mean ± SEM *n* = 5–6/group in **(A,B)** and *n* = 10–12/group in **(C–E)**. Statistical analysis was performed using one-way ANOVA and Bonferroni’s *post hoc* test. *p < 0.05, **p < 0.01, ***p < 0.001, ****p < 0.0001. Sens, sensitization; OIT, oral immunotherapy; FOS, fructo-oligosaccharides; no IT, no immunotherapy; i.g., intragastric; SCFA, short-chain fatty acids.

Bacterial fermentation of FOS leads to production of SCFA. After sensitization at D35, the mean total SCFA level was higher in the cecum of sensitized control mice than in sham-sensitized control mice (*p* = 0.017) (Figure 7A). Considering individual SCFA, a similar pattern was observed for butyric acid; however, differences between the groups were not significant (Figure 7B). The intestinal epithelium-derived factor galectin-9 was measured in serum halfway- (D50) and after immunotherapy (D63) and after challenge (D71) (Figures 7C–E). OIT + FOS increased serum galectin-9 levels at D63 (*p* = 0.0014 compared to sensitized control, *p* = 0.001 compared to FOS no IT and *p* = 0.0161 compared to OIT) (Figure 7D) and at D71 (compared to sensitized control, FOS no IT and OIT, all *p* < 0.0001) (Figure 7E).

## Discussion

Overall, our study shows that the combination of OIT and a FOS supplemented diet effectively reduces allergic symptoms upon challenge. We observed increased percentages of Foxp3+ Tregs and a reduction in T cell responsiveness and mast cell number and activation. The presence of both FOS and whey protein in the gut induces release of IEC-derived galectin-9, a factor that directly supports the process of tolerance induction *via* Treg differentiation (24). Although the exact mechanism of action has to be determined, to our knowledge, this is the first demonstration of the improved efficacy of OIT in combination with immunomodulatory food components in a murine CMA model.

In the current study, OIT with 10 mg whey induced desensitization as shown by decreased anaphylaxis symptom scores after i.d. challenge. However, the combination of OIT and FOS was the most effective approach in terms of clinical protection against challenge-induced acute symptoms of anaphylaxis and reduced activation and number of mast cells in the gastrointestinal tract. The supporting effect of FOS during OIT was evident from the reduction in the challenge-induced ear swelling in OIT + FOS mice compared to OIT mice. In line with previous results, OIT effectively increased whey-specific IgG1 and IgA in serum (submitted for publication). As demonstrated in human studies, increased allergen-specific IgG and IgA levels observed during OIT are associated with allergy protection (8, 25). The most striking difference in the humoral response in our study is the prevention of the challenge-induced increase in whey-specific IgE only observed in OIT + FOS mice.

Induction of Foxp3+ Tregs occurs in the MLN *via* CD103+ DCs and involvement of TGFβ and retinoic acid (RA) (26). Under the influence of CD103+ DCs and MLN stromal cells, gut-homing receptors are expressed on the surface of Tregs (27) which facilitate migration to the intestine (28). In the current study, a significant induction of Foxp3+ Tregs was only observed during immunotherapy in the MLN of OIT + FOS mice. Since clinical protection was observed upon challenge at a later stage of the experiment in the OIT + FOS mice, we hypothesize that the Foxp3+ Tregs traveled to other sites of action after immunotherapy. It has been described that trafficking of Tregs between multiple compartments contributes to their suppressive effect (29). In accordance, we confirmed the presence of Foxp3+ Tregs in the LP of the small intestine after immunotherapy. A trend toward an increased percentage of CD103+ DCs was observed in the LP of OIT + FOS mice after i.g. challenge. Functionality of spleen-derived Foxp3+ Tregs was shown by the loss of protection against allergic symptoms after transfer of depleted cell fractions. In addition, murine models of peanut AIT have shown that several routes of administration modulate the allergic response *via* IL-10-secreting Type 1 regulatory cells (Tr1) and TGFβ secreting LAP + Th3 Tregs (30). In the current study, we were not able to measure IL-10 producing CD4+ CD25+ Tr1 cells, but the percentage of LAP+ CD4+ Foxp3− cells was increased in the MLN of OIT + FOS mice compared to OIT alone at the time point of clinical protection. Together, the data of MLN-, LP-, and spleen-derived Tregs indicate an important role for Tregs in the protective mechanism of OIT + FOS.

The induction of Foxp3+ Tregs in the MLN of OIT + FOS mice halfway immunotherapy was accompanied by reduced antigen-specific T cell responsiveness in splenocyte cultures. Only OIT + FOS reduced IL-5 production upon whey stimulation. Reduced cytokine production may either be due to the presence of suppressive Tregs or to anergy of specific T cells during immunotherapy. With the current data, it is not possible to distinguish between the two; however, the results of the whey stimulation assay in the adoptive transfer experiment show that depletion of CD25+ cells in OIT + FOS-derived cell fractions increases responsiveness of specific T cells toward the allergen.

Mast cell suppression by Tregs *via* the OX40–OX40 ligand interaction is an important control mechanism of the allergic response (31). In the current study, *ex vivo* Treg depletion in OIT + FOS-derived cell fractions prior to transfer increased mucosal mast cell activation *in vivo*. FOS supplementation might enhance the ability of Tregs to suppress mast cell activation directly. It has been reported that CD4+ CD25+ T cells derived from HMOS-treated OVA allergic mice efficiently suppressed *in vitro* IgE-mediated mast cell degranulation (32).

The immunomodulatory activities of non-digestible oligosaccharides have been described in both human and animal studies of gastrointestinal disorders and allergies including asthma (33–35). Carbohydrate structures are recognized by specific glycan receptors (e.g., C-type lectins) present on IECs and immune cells and contribute to the orchestration of the mucosal immune response (36). Moreover, epithelial transport of non-digestible oligosaccharides was shown *in vitro* (14), indicating the possible direct interaction with lymphocytes residing in the LP. Direct modulation of human monocyte-derived DCs and induction of Foxp3+ Tregs by a mixture of short-chain galacto-oligosaccharides and long-chain FOS (scGOS/lcFOS) was shown *in vitro* (37). Galectins are soluble type lectins expressed by IECs that were identified to be involved in the immunomodulatory effects of the scGOS/lcFOS mixture in an *in vitro* setting using IECs and human peripheral blood mononuclear cells (24). In particular IEC-derived galectin-9 played a key role in Th1 and Treg polarization possibly *via* conditioning of tolerogenic DCs (24). Elevated galectin-9 levels were observed in serum of cow’s milk allergic mice after scGOS/lcFOS supplementation (38). In the current study, OIT + FOS increased serum galectin-9 levels, suggesting direct modulation of IECs and a potential role in allergy protection by OIT + FOS. FOS supplementation without exposure to whey protein (OIT) could not induce galectin-9 secretion.

The influence of whey protein on the gut environment was further supported by the SCFA analysis in the cecum content. Whey administration during sensitization increased bacterial butyric acid production in the allergic mice compared to the sham-sensitized mice, suggesting altered microbiota composition and/or abundance. In the colon, the microbiota are involved in the fermentation of both (non-digestible) carbohydrates and proteins that were not digested in the upper part of the digestive tract into, e.g., acetate, propionate, and butyrate (39). The type and quantity of the produced SCFA can be a reflection of both the bacterial- and dietary composition in the colon (40). Our findings could not confirm that the combination of OIT and FOS supplementation favors the growth of butyrate-producing bacteria in the gut. Butyrate is a major energy source for colonocytes (41) and can promote the production of RA by epithelial cells via inhibition of histone deacetylase (42). RA is converted from dietary vitamin A by retinaldehyde-dehydrogenase-2, an enzyme expressed by epithelial cells and CD103+ DCs. Increased RA levels were associated with improved tolerogenic activity of CD103+ DCs *in vitro* (33) and stimulate the differentiation of naïve T cells into Tregs (43). Oral butyrate administration *via* the drinking water of antibiotic-treated mice induced extrathymic differentiation of Foxp3+ Tregs, showing direct modulation of the immune response independent of the microbiota (44). In addition, preventive butyrate administration mimicked the high fiber diet-induced protection against allergic symptoms and reduced total IgE levels in the serum of peanut allergic mice (33). The involvement of butyrate in the tolerance-associated immune response suggests a role in the protective mechanism induced by OIT + FOS; however, since no significant differences were found, butyric acid levels cannot explain the changes in allergic- and immunologic parameters observed in the current study.

The use of immunomodulatory food components in combination with AIT is a promising strategy to combine specific- and generic modulation of the immune response. A first attempt was made by co-administration of the probiotic *Lactobacillus rhamnosus* CGMCC during OIT in peanut allergic children. The authors report sustained unresponsiveness to a food challenge in 82.1% of the treated children after 2–5 weeks off-therapy (45). However, human data concerning the additive effect of pre- and/or probiotic supplementation on the efficacy of OIT is limited.

In conclusion, we show the potency of a specific mixture of FOS to support the efficacy of OIT in cow’s milk allergic mice. Foxp3+ Tregs play a potential role in the protective effect induced by OIT + FOS; however, the exact contribution of galectin-9 and butyric acid should be further studied. In addition, future research is necessary to investigate the potential of the current strategy to improve the safety of OIT and induce long-term tolerance toward food proteins after discontinuation of therapy. Understanding the complex interplay between the gut epithelium, the microbiota, and the immune system during OIT will contribute to knowledge-based application of nutritional interventions in human food allergy trials in the future.

## Ethics Statement

Experimental procedures were approved by the Ethical Committee of Animal Research of Utrecht University and complied with the principles of good laboratory animal care following the European Directive for the protection of animals used for scientific purposes.

## Author Contributions

MV, BE, and LK designed the experiments; MD participated in the experimental procedures; MV performed data collection and analysis and drafted the manuscript. LW, JS, RP, JG, BE, and LK contributed to data interpretation and critically revised the manuscript.

## Conflict of Interest Statement

None of the authors have a competing financial interest in relation to the presented work; LK is employed by Nutricia Research and BE and JG are partly employed by Nutricia Research, Utrecht, The Netherlands.

## Abbreviations

AIT, antigen-specific immunotherapy; CMA, cow’s milk allergy; OIT, oral immunotherapy; FOS, fructo-oligosaccharides; Foxp3, forkhead box protein 3; i.g., intragastric; CT, cholera toxin; i.d., intradermal; i.p., intraperitoneal; Ig, immunoglobulin; Th, T helper; SCFA, short-chain fatty acid; IL, interleukin; Treg, regulatory T cell; mMCP-1, murine mast cell protease-1; IFNγ, interferon γ; MLN, mesenteric lymph nodes.
